# Underutilization of effective coping styles in male physicians with burnout

**DOI:** 10.1371/journal.pone.0291380

**Published:** 2023-09-08

**Authors:** Sarah A. Holzgang, Mary Princip, Aju P. Pazhenkottil, Bianca Auschra, Roland von Känel

**Affiliations:** 1 Department of Consultation-Liaison Psychiatry and Psychosomatic Medicine, University Hospital Zurich, University of Zurich, Zurich, Switzerland; 2 Cardiac Imaging, Department of Nuclear Medicine, University Hospital Zurich, Zurich, Switzerland; 3 Department of Cardiology, University Hospital Zurich, Zurich, Switzerland; University of Valencia: Universitat de Valencia, SPAIN

## Abstract

Ineffective coping is a risk factor for burnout among physicians, in whom the prevalence of burnout is high and has also increased in recent years. We examined in a cross-sectional study whether physicians with burnout show different coping styles compared with healthy controls. Male physicians (n = 60) were recruited into two groups (burnout vs. healthy). The Coping Inventory for Stressful Situations (CISS) and the Maslach Burnout Inventory (MBI) were applied. Wilcoxon rank-sum test showed group differences in two of the three coping styles, task-oriented and emotion-oriented, and also in one of the two subscales of the avoidance-oriented coping: social-diversion-oriented coping. Multiple binomial logistic regression, controlling for age, showed that lower task-oriented coping (OR = 0.38 (0.13 – 0.93), *p* = 0.048, *d* = 0.534) and lower social-diversion-oriented coping (OR = 0.33 (0.11 – 0.80), *p* = 0.024, *d* = 0.611) significantly predicted the burnout group. The findings suggest that male physicians with burnout differ from healthy controls in terms of less frequent utilization of effective coping styles. These findings could be explored for their utility in preventing burnout in future studies.

## Introduction

Physicians belong to the occupational groups that are highly affected by burnout. The prevalence of burnout in physicians is about 50% [1–4] with an increase in recent years [2, 5]. First described by Freudenberger [6], burnout is a negative affective risk state whose three core symptoms are assessed with the Maslach Burnout Inventory (MBI): feelings of emotional exhaustion, alienation and disengagement from work, and reduced ability to perform at work [7]. This definition of burnout as a consequence of job stress, that has not been successfully managed, was included into the 11th version of International Statistical Classification of Diseases and Related Health Problems (ICD-11) [8]. “Burnout is a syndrome conceptualized as resulting from chronic workplace stress that has not been successfully managed. It is characterized by three dimensions: (1) feelings of energy depletion or exhaustion; (2) increased mental distance from one’s job, or feelings of negativism or cynicism related to one’s job; and (3) a sense of ineffectiveness and lack of accomplishment. Burnout refers specifically to phenomena in the occupational context and should not be applied to describe experiences in other areas of life” is the definition provided in the ICD-11 [8].

Burnout has negative consequences on both the personal and professional level [9]. These include, for example, alcoholism and drug dependence [10, 11], depression and anxiety [12–16], poor sleep quality [17, 18], musculoskeletal pain [19], and a higher rate of suicide risk [20, 21]. In addition, there is evidence that burnout among physicians may impair professionalism, increase the risk of medical errors, lower the quality of care [22], decrease patient satisfaction [23, 24] and satisfaction with career and work [17, 25]. Furthermore, an association of physician burnout with increased chance of reducing the workload [26], increased intention to leave the job/turnover/retirement early [11, 17, 25, 27–32] and increased costs due to reduced clinical hours or early retirement [33, 34], resulting in reduced productivity [17, 35] was found.

The literature shows several risk factors for physician burnout, including high workload, lack of work-life balance, uncertainty and changes in medical care and the health care system, less autonomy and control, increase in administrative tasks and ineffective coping styles [3, 36–38]. Coping is described as an attempt on a cognitive or behavioral level to meet the taxing demands or challenges of life [39]. Three different coping styles have commonly been distinguished: task-oriented coping involves finding a solution to the problem and trying to cope with the situation. Emotion-oriented copers, however, tend to be preoccupied with the situation and react to it overwhelmingly emotionally. The third coping style, avoidance-oriented coping, involves two subscales: avoidance coping through distraction from problem situations in general or by seeking social contacts (distraction-oriented and social-diversion-oriented coping) [40]. There is evidence for using these two subscales instead of avoidance-oriented coping, as results for both the three-factor model and the four-factor model are similar [40–42]. This is also supported by the evidence that the two subscales behave in opposite ways: for instance, distraction-oriented coping was shown to correlate positively with psychiatric symptoms and somatization, whereas social-diversion-oriented coping was shown to correlate negatively with depression [43, 44]. Previous studies have identified a link between ineffective coping styles and an increased risk of burnout among physicians. Depending on the style and situation, coping can have negative effects on mental states and health or, in the opposite case, may enhance psychological well-being [37, 45–50]. Therefore, burnout symptoms often occur when coping styles are no longer effective or ineffective coping styles are chosen [47, 51].

As a novelty, this study examined two clearly separable groups, male physicians with burnout and male physicians without burnout as controls [52]. This allowed us to specifically examine the extent to which the above delineated coping styles can predict burnout in physicians, controlling for age, because age has been shown to relate to coping styles [53, 54]. The specific aim was to investigate the association between the utilization of the different coping styles between physicians with burnout and their counterparts with no burnout. Based on previous research on efficient and inefficient coping styles among physicians [37, 45–50], we hypothesized that emotion-oriented coping predicts the burnout group, as well as that task-oriented coping predicts the healthy control group. With respect to the avoidance-coping style, we intentionally included the two subscales in the analysis to examine the extent to which they also predict group membership. We assumed that distraction-oriented coping predicts the burnout group and social-diversion-oriented coping the healthy control group. These are secondary analyses, and as a consequence of the parental study and its biological outcomes (which are published elsewhere), only male physicians were studied [52]. In addition, our study took place in part during the Covid-19 pandemic, when physicians were particularly challenged and coping with stress was especially important for their wellbeing.

## Methods

### Study participants

The present analysis is a secondary analysis of a parental study on the “Effect of burnout on myocardial blood flow” with the primary aim to assess cardiovascular health in male physicians with burnout [52]. Approval was obtained from the local ethics committee Zurich (BASEC-Nr. 2018–01974). This study had a cross-sectional design. Data were collected between September 2019 and December 2021. We recruited male physicians in Switzerland through hospitals, clinics, medical associations, professional journals, and direct e-mail contact. Via flyer, physicians were informed about the study, its procedure and objectives. Participation was voluntary. Informed consent was obtained from all participants involved in the study. Interested physicians contacted the study management via e-mail and were then asked about the inclusion and exclusion criteria in a telephone interview. Participants were enrolled in the study if the inclusion and exclusion criteria were met. Inclusion criteria were: Male physician, 28–65 years of age, non-smoker for at least 5 years, burnout (according to the criteria listed below). Exclusion criteria were: Known heart disease, familial hypercholesterolemia, renal insufficiency, hypertension, diabetes, obesity, burnout or depression in the past, current depression, cognitive impairment, allergy to iodinated contrast media, contraindication to adenosine, beta blockers or isosorbide dinitrate and the participant would not like information about clinically relevant cardiac investigations. 143 potential subjects were screened, whereof 60 were included in the study. Reasons for exclusion were either that inclusion criteria were not met, exclusion criteria were present, or there was no longer interest in participating in the study. The total of 60 male participants were divided into two groups, the burnout group and the healthy control group, each with 30 participants. Using the Maslach Burnout Inventory-Human Services Survey (MBI-HSS) [55] and the Patient Health Questionnaire-9 (PHQ-9) [56], screening and classification were done to assign participants to two extreme groups. Participants were included in the burnout group based on the following cutoff values of their MBI-HSS subscores: Emotional exhaustion (EE) ≥ 27 and/or Depersonalization (DP) ≥ 10 (with min. EE ≥ 20). Complementary a PHQ-9 score ≤ 14, reflecting at the most moderate depressive symptoms, was required. For the healthy control group, the following cutoff scores for the MBI-HSS were used: EE < 16 and DP < 7 [4], and a PHQ-9 score ≤ 10, reflecting mild depressive symptoms at the most, was an additional requirement [57]. Because the third subscale of the MBI-HSS, Personal accomplishment (PA), has been shown in the literature to evolve relatively independently of the other two subscales, PA scores were not considered for the group assignment [58–60]. Since we collected the data in part during the Covid-19 pandemic, we asked participants questions about anxiety, well-being, and workload directly related to Covid-19. Ten participants were screened before the pandemic and 50 during the pandemic with no differences in Covid-19 related questions between physicians with vs. without burnout. The authors had access to information during and after the study that can be used to identify individual participants; this access is password protected.

### Instruments and outcome measures

Coping Inventory for Stressful Situations (CISS): To assess coping styles, we used the short version of the CISS [44]. The 24-item self-report questionnaire covers three different coping styles, which are task-oriented coping, emotion-oriented coping and avoidance-oriented coping; the latter consists of the two subscales distraction-oriented coping and social-diversion-oriented coping. Questions are answered on a 5-point Likert scale from 1 = “not at all” to 5 = “very much”. Typical items are “Analyze the problem before reacting” for task-oriented coping, “Blame myself for having gotten into this situation” for emotion-oriented coping, “I spend time with a special person” for social-diversion-oriented coping and “See a movie” for distraction-oriented coping. We found a good internal consistency for the task-oriented coping scale (Cronbach’s α = 0.84), the emotion-oriented coping scale (Cronbach’s α = 0.86), and the social-diversion-oriented coping subscale (Cronbach’s α = 0.82). Internal consistency was acceptable for the avoidance-oriented coping scale (Cronbach’s α = 0.75) and the distraction-oriented coping subscale (Cronbach’s α = 0.72).

Maslach Burnout Inventory (MBI): The MBI is a self-assessment questionnaire to assess burnout severity [61]. The 22-item German version of the MBI-HSS was used in this study [55]. Each of the 22 items is rated on a 7-point scale ranging from “never” to “daily”. the MBI-HSS has three subscales which are “Emotional exhaustion” (EE, 9 items), “Depersonalization” (DP, 5 items), and “Personal accomplishment” (PA, 8 items). The three subscales can be analyzed individually. The EE subscale measures the feeling of being emotionally overwhelmed and exhausted by work. The DP subscale measures an uncaring and impersonal response toward care recipients such as patients, while the PA subscale assesses feelings about competence and successful achievements at work. Typical items are “I feel used up at the end of the work day” or “I feel fatigued when I get up in the morning and have to face another day on the job” for EE, “I feel that I treat some of my clients as if they were impersonal objects” or “I worry that this job is hardening me emotionally” for DP, and “I very effectively deal with the problems of my client” or “I can easily create a relaxed atmosphere with my clients” for PA. The internal consistency was excellent for the EE subscale (Cronbach’s α = 0.94) and good for both the DP (Cronbach’s α = 0.89) and PA subscale (Cronbach’s α = 0.81).

Patient Health Questionnaire-9 (PHQ-9): To assess the severity of depressive symptoms we used the German version of the PHQ-9 which has nine items patients have to self-rate on a four-point Likert scale ranging from 0 to 3 [56]; the totals score ranges from 0 to 27. Higher scores are indicative of more severe depressive symptoms. Internal consistency of the total score was good in our sample (Cronbach’s α = 0.79).

MBI and PHQ-9 were collected by phone at screening, the CISS was answered by participants on printed questionnaires on the day of the examination. None of the participants had missing data.

### Data analysis

The present analyses are secondary analyses of the parent study “Effect of burnout on myocardial blood flow”. Therefore, both the test for sufficient statistical power and the sample size were chosen with respect to the primary endpoint of the parental study [52].

Statistical analyses were performed using R statistical software [62]. A p-value of < 0.05 was considered statistically significant. As not all data were normally distributed, group differences were calculated using non-parametric tests: Fisher’s exact test and Wilcoxon rank-sum test. Using binomial logistic regression and the z-transformed values of coping scale scores, the prediction of group membership (burnout vs. healthy) was tested depending on the coping styles, the calculated odds ratios were converted to the effect size Cohen’s d to ensure comparability. We calculated two models. The first model with the three coping styles and the second one with the first two coping styles (task-oriented and emotion-oriented) and the two subscales of the third coping style (distraction-oriented and social-diversion-oriented coping). We decided to use the second model because it showed the better fit considering the AIC (Akaike information criterion). Additionally, we controlled in the model for the variable “age”. Regression output revealed no concern for multicollinearity with variance inflation factor (VIF) < 1.6 for all variables in the model.

## Results

With the exception of age and job satisfaction, the two groups showed very similar demographic characteristics (Table 1) [52]. Both groups were very similar in terms of body mass index, marital status, job status, years working as a doctor, working hours per week, providing emergency service and night work and employment, whereas the physicians in the burnout group were significantly younger (*p* = 0.022) and showed significantly lower job satisfaction (*p* = <0.001). In terms of medical specialties, one-third were internists, just under one-fifth were surgeons, and one-tenth were psychiatrists, with no significant difference between the two groups in this regard. Expectedly, as a consequence of the formation of the two extreme groups regarding burnout severity, the two groups differed significantly with respect to the total MBI score, the scores of the three MBI subscales (emotional exhaustion, depersonalization, and personal accomplishment) and the depressive symptom score (all *p* = <0.001, Table 2).

**Table 1:** Sample characteristics.

Characteristic		Total sample, *n* = 60		Burnout, *n* = 30				Control, *n* = 30					
		n (%)	Mean (SD^2^)	n (%)	Mean (SD^2^)	Median	IQR^3^	n (%)	Mean (SD^2^)	Median	IQR^3^	*z*-value^1^	*p*-value^1^
Age (years)			49.85 (9.59)		46.77 (10.56)	45.00	18.25		52.93 (7.48)	52.00	12.00	-2.29	**0.022**
Body mass index (m2/kg)			24.99 (2.96)		25.63 (3.09)	25.25	3.29		24.35 (2.72)	23.92	2.90	1.75	0.800
Marital status	married	44 (73%)		21 (70%)				23 (77%)					0.771
	other	16 (27%)		9 (30%)				7 (23%)					
Job status	full time	48 (80%)		25 (83%)				23 (77%)					0.748
	part time	12 (20%)		5 (17%)				7 (23%)					
Years working as a doctor			21.71 (9.97)		19.08 (10.97)	17.50	17.75		24.33 (8.23)	22.50	13.50	-1.92	0.055
Working hours per week	≤ 42.5 hours	7 (12%)		2 (6.7%)				5 (17%)					0.288
	42.6–50 hours	14 (23%)		9 (30%)				5 (17%)					
	> 50 hours	39 (65%)		19 (63%)				20 (67%)					
Providing emergency service		42 (70%)		22 (73%)				20 (67%)					0.779
Night work		35 (58%)		18 (60%)				17 (57%)					1.000
Employment	self-employed	20 (33%)		10 (33%)				10 (33%)					1.000
	hospital	38 (63%)		19 (63%)				19 (63%)					
	self-employed and hospital	2 (3.3%)		1 (3.3%)				1 (3.3%)					
Job satisfaction	very dissatisfied	1 (1.7%)		1 (3.3%)				0 (0%)					**<0.001**
	dissatisfied	1 (1.7%)		1 (3.3%)				0 (0%)					
	partly satisfied, partly dissatisfied	14 (23%)		14 (47%)				0 (0%)					
	satisfied	21 (35%)		11 (37%)				10 (33%)					
	very satisfied	23 (38%)		3 (10%)				20 (67%)					
Medical specialty	Psychiatry	6 (10%)		2 (6.7%)				4 (13.3%)					0.175
	Cardiology	3 (5%)		1 (3.3%)				2 (6.7%)					
	Internal medicine	20 (33%)		12 (40%)				8 (27%)					
	Oncology	4 (6.7%)		0 (0%)				4 (13%)					
	Surgery	11 (18.3%)		4 (13.3%)				7 (23.3%)					
	Neurology	3 (5%)		2 (6.7%)				1 (3.3%)					
	Other	13 (22%)		9 (30%)				4 (13.3%)					

^1^ Wilcoxon rank-sum test, Fisher’s exact test

^2^ Standard deviation

^3^ Interquartile range

**Table 2:** Descriptive statistics of depression, maslach burnout inventory.

Variables		Total sample, *n* = 60	Burnout, *n* = 30			Control, *n* = 30				
		Mean (SD^2^)	Mean (SD^2^)	Median	IQR^3^	Mean (SD^2^)	Median	IQR^3^	*z*-value^1^	*p*-value^1^
Depressive symptoms (PHQ-9)		6.27 (4.21)	9.4 (2.69)	9.00	3.75	3.13 (2.92)	2.00	3.00	5.67	**< 0.001**
Maslach Burnout Inventory (MBI)^4^	Total score	1.68 (1.11)	2.68 (0.57)	2.62	0.91	0.68 (0.33)	0.71	0.50	6.65	**< 0.001**
	Emotional Exhaustion	19.53 (12.78)	31.13 (5.84)	30.50	8.75	7.93 (4.43)	7.00	6.75	6.66	**< 0.001**
	Depersonalization	8.05 (7.26)	13.77 (6.08)	12.00	8.75	2.33 (1.67)	2.00	2.00	6.45	**< 0.001**
	Personal accomplishment	8.68 (5.58)	12.43 (4.61)	12.00	6.75	4.93 (3.6)	5.50	5.00	5.45	**< 0.001**

^1^ Wilcoxon rank-sum test

^2^ Standard deviation

^3^ Interquartile range

^4^ Screening and classification for group assignment was done with the MBI [55]

Table 3 shows the group differences regarding the different coping styles: in the group with burnout, task-oriented coping was significantly lower (*p* = 0.001) and emotion-oriented coping was significantly higher than in the group without burnout (*p* = <0.001). Avoidance-oriented coping showed no significant group difference (*p* = 0.328). However, regarding its two subscales, physicians with burnout showed significantly less social-diversion-oriented coping than physicians without burnout (*p* = 0.001). No significant group difference was seen for the distraction-oriented coping subscale (*p* = 0.096).

**Table 3:** Wilcoxon rank-sum test testing for group differences in Coping Inventory for Stessful Situations (CISS).

Variables		Total sample, *n* = 60	Burnout, *n* = 30			Control, *n* = 30					
		Mean (SD^2^)	Mean (SD^2^)	Median	IQR^3^		Mean (SD^2^)	Median	IQR^3^	*z*-value^1^	*p*-value^1^
Coping Inventory for Stressful Situations (CISS)	Task-oriented coping	3.88 (0.58)	3.62 (0.54)	3.75	0.78		4.13 (0.52)	4.00	0.81	-3.30	**0.001**
	Emotion-oriented coping	2.47 (0.81)	2.90 (0.78)	2.94	1.09		2.04 (0.58)	1.88	0.84	4.15	**<0.001**
	Avoidance-oriented coping	2.82 (0.70)	2.72 (0.65)	2.81	0.94		2.92 (0.74)	2.88	0.75	-0.98	0.328
	Distraction-oriented coping	2.38 (0.89)	2.52 (0.75)	2.50	0.88		2.23 (1.01)	1.88	1.19	1.67	0.096
	Social-diversion-oriented coping	3.26 (0.90)	2.92 (0.83)	2.88	1.25		3.60 (0.84)	4.00	1.19	-3.22	**0.001**

^1^ Wilcoxon rank-sum test

^2^ Standard deviation

^3^ Interquartile range

The multiple binomial logistic regressions were calculated to test the prediction of group membership (burnout vs. healthy) by coping styles controlling for age. Table 4 shows that task-oriented coping (*p* = 0.048, *d* = 0.534) and social-diversion-oriented coping (*p* = 0.024, *d* = 0.611) were significantly predictive of group membership. The less task-oriented and the less social-diversion-oriented coping the more likely a participant belonged to the burnout group. Emotion-oriented coping (*p* = 0.137, *d* = 0.406) and distraction-oriented coping (*p* = 0.153, *d* = 0.289) were not significantly predictive of group membership.

**Table 4:** Multiple binomial logistic regression analysis to predict burnout vs. control by coping styles (CISS) controlled for age.

	Group					
Predictors	Odds Ratios	std. Error	95% CI	z	*p*	*d*
(Intercept)	1.15	0.42	0.57 – 2.41	0.38	0.704	0.077
Task-oriented coping	0.38	0.19	0.13 – 0.93	-1.98	**0.048**	0.534
Emotion-oriented coping	2.09	1.04	0.82 – 5.98	1.49	0.137	0.406
Distraction-oriented coping	1.69	0.62	0.84 – 3.63	1.43	0.153	0.289
Social-diversion-oriented coping	0.33	0.16	0.11 – 0.80	-2.25	**0.024**	0.611
Age	0.47	0.18	0.20 – 0.96	-1.92	0.055	0.416
Observations	60					
R^2^ Tjur	0.457					

## Discussion

In this study, we examined differences in coping styles between male physicians with burnout and their healthy counterparts without burnout. Two of three coping styles and one of two subscales showed significant group differences. Specifically, male physicians with less utilization of both task-oriented and social-diversion-oriented coping were more likely to have burnout, whereas emotion-oriented and distraction-oriented coping styles did not predict group membership. We interpret this, in particular, as the underutilization of the efficient coping styles task-oriented coping and social-diversion-oriented coping playing an important role with regard to burnout among physicians.

Our finding on male physicians with and without burnout associated with different coping styles are consistent with previous research. Previous studies on physicians showed an association between the inefficient emotion-oriented coping style and a higher risk of burnout in physicians, whereas the effective task-oriented coping style was shown to be associated with a lower burnout risk [46–50, 63]. Our results add to these findings. Low task-oriented coping was predictive for the burnout group in our study, although emotion-oriented coping was not, which may be a consequence of controlling our analysis for age. Age was significantly lower in the burnout group than in the healthy control group. Emotion-oriented coping was significantly more utilized by physicians with burnout, implying that young male physicians with burnout are more likely to engage in emotion-oriented coping. As studies have shown that age per se does not tend to be a predictor of emotion-oriented coping [54] our finding might also reflect less professional experience in younger physicians. The observation that emotion-oriented coping style did not predict group membership may also be related to the fact that we studied only male physicians. Studies with adults have shown that men use the emotion-oriented coping style less than women [40, 64].

With regard to avoidance-oriented coping, there are also conflicting study results. In some studies, no correlations were found with burnout [48], although in others there were negative correlations with MBI EE and positive correlations with PA [50] and with DP [49]. Other studies have shown positive associations between avoidance-oriented coping and burnout, respectively psychological and physical conditions [65, 66].

These differing results may be related in part to the fact that avoidance-oriented coping is composed of a more effective (social-diversion-oriented) and a more ineffective (distraction-oriented) coping style, which might explain studies attempting to interpret them separately [40, 41, 43, 44]. We were able to address this conceptual issue by including these two subscales from the CISS for our analysis. We observed that the social-diversion-oriented coping style was less likely to be used by the burnout group than by the healthy control group. It is possible, for example, that physicians experiencing burnout may increasingly withdraw from social activities, making coping through social distraction less of an option.

We could not verify a group difference for distraction-oriented coping, nor was this subscale predictive of group membership. Looking at the mean scores values of the two groups, it is noticeable that both groups gave the answer "rather atypical" to the questions about this coping style. This, in turn, could be related to the physicians’ experience that it is difficult to cope by distraction in the hectic everyday work, considering that the questions asked about distracting coping behaviors like shopping and watching movies.

These findings of our observational study may have practical implications for future intervention studies to prevent burnout in male physicians. The results of the multiple binomial logistic regression analysis suggest that, on the one hand, effective coping styles could particularly be promoted, as they significantly predicted group membership of the healthy controls, which is also consistent with previous findings [67]. On the other hand, it could also be important to reduce the utilization of ineffective coping styles [68, 69]. Intervention studies may want to specifically examine the extent to which promoting task-oriented and social-diversion-oriented coping can reduce burnout risk among physicians. This could also be examined in terms of reducing emotion-oriented coping in early career physicians.

## Strengths and limitations

To our best knowledge, this is the first study to examine two well-separated groups of physicians with and without burnout in terms of the predictive power of coping styles; yet the study has also noteworthy limitations. Study participants were recruited with the aim to form two predefined extreme groups in terms of burnout severity. Therefore, we were unable to analyze continuous scores of the MBI. The generalizability of our findings is limited due to several reasons. Due to the study design of the parent study “Effect of burnout on myocardial blood flow”, the sample was small compared to other studies investigating coping styles. Furthermore, only male physicians were studied to keep the number of confounders regarding the primary endpoints published elsewhere (e.g., hormonal influences on cardiovascular health) as low as possible. In addition, the study was conducted in Switzerland. Male physicians participated in our study voluntarily and of their own interest, hence the possibility of self-selection cannot be precluded. Thus, whether the results and their implications would be the same for female physicians and in other countries remains unclear. Also, the study was conducted during the Covid-19 pandemic when physicians were particularly challenged, which may have reduced their capacity to participate in a study. Utilizing certain coping styles might have been difficult due to pandemic restrictions, including social distraction. The cross-sectional data of our study do not allow causal conclusions as to whether specific coping styles are more likely to lead to burnout or vice versa.

## Conclusions

Our findings suggest that effective coping styles are underutilized by physicians with burnout and that effective coping styles are more likely to be used by physicians without burnout, which is consistent with previous research. Especially underutilization of the effective coping styles, task-oriented coping and social-diversion-oriented coping, might play an important role with regard to burnout among male physicians. To substantiate these findings, longitudinal studies with larger samples, including female physicians, and interventions studies targeting an increase in effective coping styles and a reduction in ineffective coping styles are needed.
